# Prediction of giant and ideal Rashba-type splitting in ordered alloy monolayers grown on a polar surface

**DOI:** 10.1093/nsr/nwaa241

**Published:** 2020-09-25

**Authors:** Mingxing Chen, Feng Liu

**Affiliations:** Key Laboratory for Matter Microstructure and Function of Hunan Province, Key Laboratory of Low-Dimensional Quantum Structures and Quantum Control of Ministry of Education, Synergetic Innovation Center for Quantum Effects and Applications (SICQEA), School of Physics and Electronics, Hunan Normal University, Changsha 410081, China; Department of Materials Science and Engineering, University of Utah, Salt Lake City, UT 84112, USA

**Keywords:** Rashba effect, interface, spin-orbit coupling

## Abstract

A large and ideal Rashba-type spin-orbit splitting is desired for the applications of materials in spintronic devices and the detection of Majorana fermions in solids. Here, we propose an approach to achieve giant and ideal spin-orbit splittings through a combination of ordered surface alloying and interface engineering, that is, growing alloy monolayers on an insulating polar surface. We illustrate this unique strategy by means of first-principle calculations of buckled hexagonal monolayers of SbBi and PbBi supported on Al_2_O_3_(0001). Both systems display ideal Rashba-type states with giant spin-orbit splittings, characterized with energy offsets over 600 meV and momentum offsets over 0.3 Å^−1^, respectively. Our study thus points to an effective way of tuning spin-orbit splitting in low-dimensional materials to draw immediate experimental interest.

## Teaser

A strategy that combines ordered surface alloying and polar surface is proposed to achieve giant and ideal Rashba splittings in buckled monolayers.

## INTRODUCTION

The Rashba effect is referred to as the spin-orbit (SO) splitting at surfaces/interfaces due to the broken inversion symmetry [1,2], which has led to many exotic quantum phenomena and novel applications, ranging from the spin Hall effect, Majorana fermions in solids and the spin field-effect transistor [3–5]. The effective Rashba Hamiltonian for an electron with momentum $\mathbf {k}$ and spin $\mathbf {\sigma }$ can be written as

$$\begin{equation*}
\hat{H}_{R} = \lambda \mathbf {\sigma } \cdot (\mathbf {E_z}\times \mathbf {k}),
\end{equation*}$$

where λ is the strength of the SO coupling (SOC) and $\mathbf {E_z}$ is the electric field perpendicular to the surface/interface created by a perpendicular potential gradient related to the structural asymmetry. The SO splitting is defined as α_*R*_ = 2*E*_*R*_/*k*_*R*_, where *E*_*R*_ and *k*_*R*_ are the Rashba energy and momentum offset, respectively.

In the ongoing exploration of large Rashba-type SO splittings in materials, enhancing the strength of λ by introducing heavy elements has been extensively used [6–11]. In particular, the ordered ($\sqrt{3}\times \sqrt{3}$) superstructure of Bi/Ag(111) in which one surface Ag is replaced by Bi displays a giant Rashba energy offset (*E*_*R*_) of 200 meV and momentum offset (*k*_*R*_) of 0.13 Å^−1^ [6]. In addition, semiconducting substrates are used to avoid the mixing of the Rashba states and the spin-degenerate substrate states, in order to create so-called ideal Rashba states [12–22]. On the other hand, the interfacial dipole field can be used to enhance $\mathbf {E_z}$ and hence the SO splitting. Polar semiconductors are effective substrates to serve this purpose [22–27]. In fact, the surfaces of polar semiconductors bismuth tellurohalides BiTeX (X = Cl, Br and I) have been found to exhibit giant SO splittings [28,29]. Moreover, the Rashba SO splitting can be controlled by the electric polarization in ferroelectric materials and substrates [23–25,30–32]. Despite these achievements, natural materials exhibiting both giant and ideal Rashba states are rare. Therefore, artificial interfaces by *a priori* theoretical design are highly desirable to fill this outstanding gap.

In this work, we demonstrate the design principle to create giant and ideal interfacial Rashba states by combining ordered surface alloying and growth on a polar insulator/semiconductor surface. Using density-functional theory (DFT) calculations, we show unprecedented large Rashba energy offsets over 600 meV and momentum offsets over 0.3 Å^−1^ for both SbBi/Al_2_O_3_(0001) and PbBi/Al_2_O_3_(0001), which are roughly three times of those in Bi/Ag(111). Also, such Rashba states are ideally situated inside the band gap of Al_2_O_3_(0001).

## RESULTS

**Figure 1.**
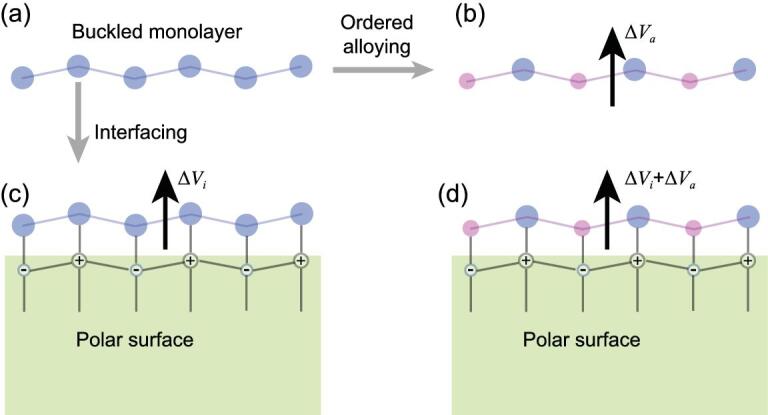
Sketch of manipulating the potential gradient through a buckled monolayer by ordered alloying and interfacing. (a) The geometry of a free-standing buckled monolayer. (b) Perpendicular potential gradient (Δ*V*_*a*_) induced by ordered alloying. (c) Interface-induced potential gradient (Δ*V*_*i*_) through the monolayer when placing it onto a polar surface, e.g. growing it on a substrate. A large Δ*V*_*i*_ can be favored when they form a special interface structure such that the two atoms in the monolayer bind to different types of ions in a polar surface. (d) The combined effect of the ordered alloying and interfacing. The arrows indicate the perpendicular potential gradients. Different types of atoms are in different colors. The +/− symbols represent cations/anions in the polar surface.

We begin by illustrating the general idea as shown in Fig. 1. Our structural model makes use of the geometric and electronic properties of both the buckled overlayer and substrate. The buckled honeycomb structure exists in a number of elemental layered materials, such as silicene, germanene, stanene and Bi(111) monolayers [33–37], which consist of two trigonal sublattices sitting at different heights. Now imagine that these two sublattices are made of different types of atoms to break inversion symmetry. Then a Rashba SO splitting will arise in such a buckled honeycomb alloy monolayer. Apparently, to enhance SOC, the optimal choice to form the monolayer are heavy atoms, such as Bi, Pb and Sb. This constitutes our first idea of the surface alloying effect. Next, let us imagine growing this alloy monolayer on a polar insulator/semiconductor substrate. The polar surface induces an additional perpendicular potential gradient (Δ*V*_*i*_) through the alloy monolayer. If Δ*V*_*i*_ is along the same direction as the alloying induced potential gradient Δ*V*_*a*_, then it will further enhance Δ*V*_*a*_ (see Fig. 1(d)). Consequently, the combined effects of surface alloying and the polar surface conspire, leading to a giant SO in the monolayer. By properly choosing the monolayer-substrate material combinations, we can further tune the relative positions of monolayer SO states relative to that of the substrate band gap to achieve ideal Rashba-type states.

To validate our idea we performed DFT calculations for buckled monolayers of Bi (Bi-1L), Pb (Pb-1L), Sb and their ordered alloys on Al_2_O_3_(0001). Al_2_O_3_(0001) is chosen for several reasons. First, it has been extensively used as a substrate for the growth of various materials. Second, there are two different types of atoms in the surface, i.e. Al and O, which behave chemically different. In addition, the surface Al atom is slightly higher (∼0.15 Å) than the O atom, which is beneficial for growing the buckled monolayers. Therefore, we may expect an enhanced SO splitting when two different atoms in the overlayer bind to Al and O. Moreover, it has a large band gap, which is favorable for forming ideal Rashba states.

**Figure 2.**
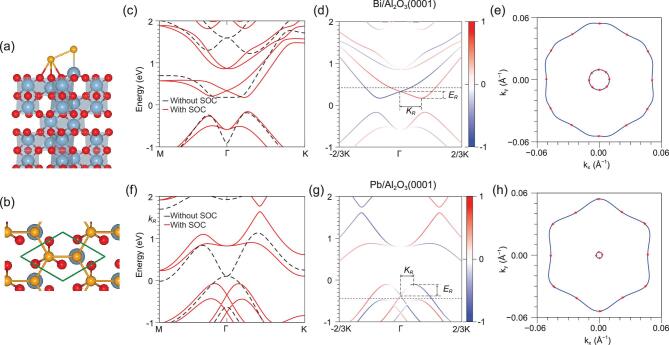
SO splittings in Bi/Al_2_O_3_(0001) and Pb/Al_2_O_3_(0001). (a, b) Side and top views of the monolayer supported on Al_2_O_3_(0001). The Al and O atoms are denoted by silver and red balls, respectively. The green box in (b) represents the primitive cell of the interface structure. (c, f) The band structures of Bi/Al_2_O_3_(0001) and Pb/Al_2_O_3_(0001), respectively. The red solid lines denote the bands derived from SOC calculations, whereas the black dashed lines represent the bands from nonrelativistic calculations. (d) Spin projections onto the direction vector of **K** × **e**_$z$_ for bands along −K-Γ−K, where **e**_$z$_ = (0, 0, 1), for Bi/Al_2_O_3_(0001). Blue and red represent positive and negative spin polarizations, respectively. (e) The Fermi surfaces for the energy marked in (b) by the dashed line. (g, h) Corresponding plots for Pb/Al_2_O_3_(0001). The red arrows in (e) and (h) denote the spin polarizations. We mark *E*_*R*_ and *k*_*R*_ for the bands discussed in the text. The Fermi level is set to zero.

We have systematically evaluated the structures and energetics of our systems. The low-energy structures were derived from our previous calculations of stanene/Al_2_O_3_(0001) using the CALYPSO structure prediction method [38], which produced the same structure for stanene/Al_2_O_3_(0001) as reported in [39]. For the homonuclear monolayers, the structure is shown in Fig. 2. The overlayers preserve the buckled honeycomb structure upon geometric relaxation and have a strong binding with the substrate. The structural properties and energetics are given in Fig. S1.

We first discuss the interfacing effect on the SO splitting in homonuclear monolayers, i.e. Bi/Al_2_O_3_(0001) and Pb/Al_2_O_3_(0001). In Fig. 2 we show the band structures for Bi/Al_2_O_3_(0001) and Pb/Al_2_O_3_(0001) with and without SOC, respectively, which reveals that both are semiconductors with a gap of about 0.30 eV when SOC is included. The most prominent feature is the large SO splittings in the overlayer due to the presence of a substrate compared to those of the free-standing monolayers [36,37,40]. For Bi/Al_2_O_3_(0001), the conduction band shows a Rashba-like splitting. While for Pb/Al_2_O_3_(0001), the Rashba-like splitting appears in the valance band. For Pb/Al_2_O_3_(0001), there are two series of Rashba-type bands mixed near Γ. They become distinct by increasing the layer distance (Fig. S2). Orbital projections reveal that the bands near the gap are basically contributed by the *p* orbitals of Bi (Pb) (Fig. S3). Thus, the Rashba states are ideal in Bi/Al_2_O_3_(0001) and Pb/Al_2_O_3_(0001).

We have checked the spin texture of the Rashba states by plotting spin projections onto the direction vector of **K** × **e**_$z$_ for the bands of the two systems, where **K** = (1/3, 1/3, 0) and **e**_$z$_ = (0, 0, 1) is the unit vector normal to the surface. The in-plane component perpendicular (parallel/antiparallel) to the vector is denoted by *S*_⊥_ (*S*_∥_), while the out-of-plane component is denoted by *S*_$z$_. In Fig. 2(b) and (e) we show the bands weighted by *S*_∥_ along −K-Γ−K, and in Fig. 2(c) and (f) we show the Fermi surface and spin texture for the energy marked in (b) and (c), respectively. For the conduction band of Bi/Al_2_O_3_(0001), *S*_$z$_ and *S*_⊥_ are negligible, confirming the Rashba nature of this band, although the outer branch undergoes a slight warping compared with the inner branch. For the valance band of Pb/Al_2_O_3_(0001), the warping becomes more prominent and *S*_$z$_ is relatively more appreciable than that for Bi/Al_2_O_3_(0001). The warping indicates that the SO splitting is anisotropic, which has the largest value along Γ–K. We marked the Rashba energy offset *E*_*R*_ and momentum offset *k*_*R*_ in Fig. 2(b) and (e). For Bi/Al_2_O_3_(0001), *E*_*R*_ is about 160 meV, comparable to that for Bi/Ag(111) (200 meV). Such an SO splitting is one order of magnitude larger than that for the pure Bi(111) (10 meV) [41]. While *k*_*R*_ is about 0.2 Å^−1^, which is 50% larger than that for Bi/Ag(111). For Pb/Al_2_O_3_(0001), *E*_*R*_ is about 260 meV, which is about 30% larger than that for Bi/Ag(111). The enhancement in *E*_*R*_ leads to a larger α_*R*_ for Pb/Al_2_O_3_(0001) compared to Bi/Ag(111), since *k*_*R*_ is basically the same for both.

**Figure 3.**
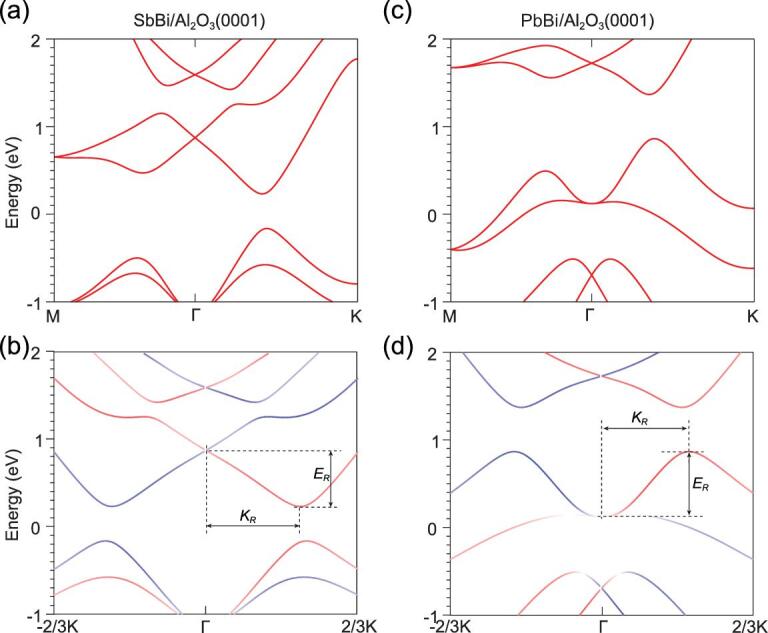
SO splittings in ordered alloy monolayers on Al_2_O_3_(0001). (a, c) Band structures of SbBi/Al_2_O_3_(0001) and PbBi/Al_2_O_3_(0001), respectively. (b, d) Corresponding spin projections onto the direction vector of **K** × **e**_$z$_ for bands along −K-Γ–K. The Fermi level is set to zero.

The large SO splitting can be further enhanced by appropriate ordered alloying. We substitute one Bi in Bi-1L by an Sb atom, which allows us to obtain a semiconducting monolayer, while replacing one Bi by Pb leads to split states crossing the Fermi level. The band structures for the lowest-energy structure for SbBi/Al_2_O_3_(0001) and PbBi/Al_2_O_3_(0001) are shown in Fig. 3. Note that the splitting is now strongly anisotropic compared to that for homonuclear monolayers. For SbBi/Al_2_O_3_(0001), the bands are pretty much similar to those for Bi/Al_2_O_3_(0001). However, *E*_*R*_ is increased to about 640 meV and *k*_*R*_ is about 0.36 Å^−1^ for the conduction band along Γ–K. Likewise, we obtain a giant SO splitting for PbBi/Al_2_O_3_(0001), for which the energy offset is as large as 740 meV and *k*_*R*_ is about 0.34 Å^−1^ for the bands crossing the Fermi level.

The large SO splitting is further confirmed by hybrid density-functional calculations (see Fig. S4). We further demonstrate that the large SO splittings are maintained even when the chemical composition is not ideal. To show this effect, we have performed calculations for Sb_1.1_Bi_0.9_/Al_2_O_3_(0001) and Sb_0.9_Bi_1.1_/Al_2_O_3_(0001) using the virtual crystal approximation to mimic a random alloy. Our calculations reveal that a 10% deviation from the ideal chemical composition has only a minor effect on the SO splitting (see Fig. S5). Moreover, to see the effect of the buckling height on the SO splitting, we carried out two additional calculations for SbBi/Al_2_O_3_(0001) with two different buckling heights. The structures were obtained by artificially adjusting the $z$ value of Sb by ±0.1 Å, which gives about an 8% change in the buckling height. As a result, *E*_*R*_ and *k*_*R*_ change by about 10% (see Fig. S6). However, we may expect that the buckling height in an experimentally grown sample should be rather close to the optimized value by our calculations.

In Table 1, we summarize the Rashba parameter α_*R*_, energy offset *E*_*R*_ and momentum offset *k*_*R*_ for the systems studied. Here *E*_*R*_ and *k*_*R*_ are for the bands marked in Figs 2 and 3. We also show the SO splitting parameters of the heavy-element doped nonpolar surface Bi/Ag(111) and the surface of polar semiconductor BiTeI for comparison. For the alloy systems SbBi/Al_2_O_3_(0001) and PbBi/Al_2_O_3_(0001), *E*_*R*_ is over 640 meV along –K-Γ–K, which is more than three times that of Bi/Ag(111) and six times that of the surface of BiTeI. While *k*_*R*_ is almost three times that of Bi/Ag(111) and one order of magnitude larger than BiTeI. Consequently, an unprecedentedly large Rashba parameter α_*R*_ is obtained for both SbBi/Al_2_O_3_(0001) and PbBi/Al_2_O_3_(0001). Compared to the isolated system, e.g. SbBi, interfacing with Al_2_O_3_(0001) enhances *E*_*R*_ by a factor of three and enhances α_*R*_ by a factor of roughly two. Our results thus demonstrate that the combination of ordered alloying and interfacing with a polar surface can be an effective strategy to obtain a giant SO splitting in buckled monolayers.

**Table 1.** SO splitting in buckled monolayers on Al_2_O_3_(0001) and selected materials from the literature. Here *E*_*R*_ and *k*_*R*_ are the Rashba energy offset and momentum offset for the bands marked in Figs 2 and 3, and α_*R*_ is the Rashba parameter calculated by α_*R*_ = 2*E*_*R*_/*k*_*R*_. The identity indicates whether the Rashba-type states mix with the substrate states.

	*E*_*R*_	*k*_*R*_	α_*R*_		
Materials	(meV)	(Å^−1^)	(eV Å)	Identity	Reference
					
**Heavy-element doped nonpolar surface**					
Bi/Ag(111)	200	0.13	3.05	Mixed	[6]
**Polar surface**					
BiTeI	108	0.05	4.30	Ideal	[29]
**Homolayer on polar surface**					
Au/InSe(0001)	–	–	0.45	Ideal	[22]
Bi/Al_2_O_3_(0001)	160	0.20	1.61	Ideal	This work
Pb/Al_2_O_3_(0001)	266	0.13	4.14	Ideal	This work
**Isolated ordered alloy monolayer**					
SbBi	150	0.21	1.40	Ideal	This work
**Ordered alloy monolayer on polar surface**					
SbBi/Al_2_O_3_(0001)	641	0.36	3.55	Ideal	This work
PbBi/Al_2_O_3_(0001)	741	0.34	4.38	Ideal	This work

The large SO splittings originate from the special geometry that induces a large perpendicular potential gradient through the overlayer. Within the tight-binding approximation, the effect of the perpendicular potential gradient goes into on-site Hamiltonian matrix elements. We perform analyses on the Hamiltonian matrix elements as derived from the linear combination of the atomic orbital calculation [42], which reproduced the band structures shown in Figs 2 and 3 (see Fig. S7). In Table 2 we list the differences in the on-site Hamiltonian matrix elements for the *p* orbital between the two atoms in the overlayer, denoted as Δ*H*_α, α_, for which the atom binding to the oxygen atom is taken as the reference. For the freestanding Bi-1L and Pb-1L, the Δ*H*_α, α_ are zeros, which become greater than 0.5 eV when supported on Al_2_O_3_(0001). The interface-induced perpendicular electric field through the supported Bi-1L is about 0.5 eV/Å, estimated by $\Delta H_{p_z,p_z}$/Δ$z$. The estimated *E*_$z$_ for Pb-1L is double that of Bi-1L. This trend is consistent with the fact that α_*R*_ for Pb/Al_2_O_3_(0001) is much larger than that for Bi/Al_2_O_3_(0001) (see Table 1). The difference between $\Delta H_{p_x,p_x}$ ($\Delta H_{p_y,p_y}$) and $\Delta H_{p_z,p_z}$ is attributed to the two-dimensional nature of the interface structure.

**Table 2.** Effects of alloying and interfacing on the on-site hopping parameters (*H*_α, α_) for the monolayers on Al_2_O_3_(0001). Here Δ*H*_α, α_ is the difference in the on-site hopping terms for orbital α between the two atoms in the overlayer, which takes the atom binding to the oxygen atoms as the reference, and Δ$z$ denotes the buckling in the overlayer.

	$\Delta H_{p_x,p_x}$	$\Delta H_{p_y,p_y}$	$\Delta H_{p_z,p_z}$	Δ$z$
Materials	(eV)	(eV)	(eV)	(Å)
Pb/Al_2_O_3_(0001)	0.83	0.83	1.32	1.30
Bi/Al_2_O_3_(0001)	0.58	0.58	0.73	1.50
Isolated SbBi	0.20	0.20	0.68	1.25
SbBi/Al_2_O_3_(0001)	1.07	1.07	1.31	1.25
Isolated PbBi	−0.46	−0.46	−0.68	1.37
PbBi/Al_2_O_3_(0001)	0.37	0.37	0.64	1.37

For the ordered alloy systems SbBi and PbBi, the structure naturally gives a difference in the on-site Hamiltonian matrix elements, which results in SO splittings in the electronic bands (Fig. S8). From Table 2 we can see that the Δ*H*_α, α_ are enhanced by over 0.6 eV for the supported SbBi. For PbBi, interfacing reverses the potential gradient, which leads to changes in Δ*H*_α, α_ over 0.8 eV for the *p* orbital. Consequently, the SO splitting is significantly enhanced by interfacing compared to that for the isolated monolayer alloy.

In addition to the giant SO splittings, some of our systems may have nontrivial topological properties. We have calculated the evolution of the Wannier function center based on the method proposed in [43]. Our results reveal that Pb/Al_2_O_3_(0001) and Bi/Al_2_O_3_(0001) have a *Z*_2_ equal to one (Fig. S9), whereas SbBi/Al_2_O_3_(0001) has a *Z*_2_ equal to zero (not shown). We further performed calculations of edge states by making the surface monolayer into a nanoribbon. Our calculations show that there are gapless edge states for Pb/Al_2_O_3_(0001) and Bi/Al_2_O_3_(0001) (Fig. S9). Thus, these two systems are expected to be topologically nontrivial.

Lastly, we discuss the experimental feasibility of our systems. The layered crystal structure of bismuth favors the growth of Bi-1L, which has been obtained on several semiconducting substrates such as Bi_2_Te_3_ and Bi_2_Se_3_ by molecular beam epitaxy (MBE) growth [44–46]. While the buckled honeycomb structure of Pb was predicted to be energetically lower than the planar one [40]. Alloy systems such as Sb_1−*x*_Bi_*x*_ and Pb_1−*x*_Bi_*x*_ have been grown on Ag(111) [8–10]. Moreover, Pb-based and Bi-based ordered alloys on surfaces, e.g. Tl_3_Pb/Si(111) and Sn_2_Bi/Si(111), have also been obtained in recent experiments [18,21]. Regarding the substrate, Al_2_O_3_(0001) has been extensively used as a substrate for the growth of thin films. For instance, it has been used for the growth of silicene [47], confirming our early DFT prediction [48]. Our calculations show that the binding energy (*E*_*b*_) is about 0.25 eV/Bi for Bi/Al_2_O_3_(0001), much larger than that for Bi/Bi_2_Te_3_ (0.10 eV/Bi), a system already obtained in the laboratory [44]. While for Pb/Al_2_O_3_(0001) and the alloy systems, *E*_*b*_ is larger than 0.50 eV/atom, favoring the monolayer structure. For isolated SbBi, a previous study found that it is both dynamically and thermodynamically stable [20]. We further performed an *ab initio* molecular dynamics simulation (*T* = 500 K) for the supported system, i.e. SbBi/Al_2_O_3_(0001), which shows that the structure is also thermodynamically stable (Fig. S10). Therefore, the growth of our systems can be highly feasible.

## CONCLUSION

In summary, we have proposed a strategy that combines the surface alloying and interface engineering to manipulate the SO splitting in two-dimensional materials. We have illustrated the idea in low-buckled hexagonal monolayers, e.g. Bi-1L, Pb-1L and their alloys SbBi and PbBi, supported on Al_2_O_3_(0001) by DFT calculations. Our calculations show giant Rashba-like SO splittings in these interface structures. In particular, the Rashba energies and momentum offsets of the split states for SbBi/Al_2_O_3_(0001) and PbBi/Al_2_O_3_(0001) are roughly three times those of Bi/Ag(111). Our study thus provides an effective way of manipulating the SO splitting in layered two-dimensional materials for potential applications in spintronics and the study of Majorana fermions in solids.

## METHODS

Our calculations were performed using the Vienna Ab Initio Simulation Package [49]. The interface structure was modeled in terms of a repeated slab, separated from its periodic images by 10 Å vacuum regions. We note that the lattice mismatch strain can be effectively relieved by adjusting the buckling height of the overlayer [45,46]. Therefore, the interface structure in our modeling contains only one unit cell for both the overlayer and the substrate. The pseudopotentials were constructed by the projector augmented wave method [50]. Van der Waals (vdW) dispersion forces between the adsorbate and the substrate were accounted for through the optimized Perdew–Burke–Ernzerhof density functional by using the vdW density-functional method [51]. A 11×11 Γ-centered Monkhorst-Pack *k*-point mesh was used to sample the surface Brillouin zone. A plane-wave energy cutoff of 400 eV was used for all the calculations. The overlayer atoms and the surface Al and O atoms were fully relaxed until the residual forces were less than 0.001 eV/Å.

## Supplementary Material

nwaa241_Supplemental_File
