# Supplementary material for: Prediction of giant and ideal Rashba-type splitting in ordered alloy monolayers grown on a polar surface
Source: Natl Sci Rev. 2020 Sep 25;8(4):nwaa241. doi: 10.1093/nsr/nwaa241 (PMC8288359; doi:10.1093/nsr/nwaa241)
Supplement: nwaa241_Supplemental_File [file nwaa241_supplemental_file.pdf]

Prediction of giant and ideal Rashba-type splitting in ordered alloy monolayers grown on a polar surface: Supplemental Materials

1. Structures and energetics for monolayers on Al_2O_3(0001)

The low-lying energy structures for Bi-1L, Pb-1L and their alloys on Al_2O_3(0001) are plotted in Fig. S1.

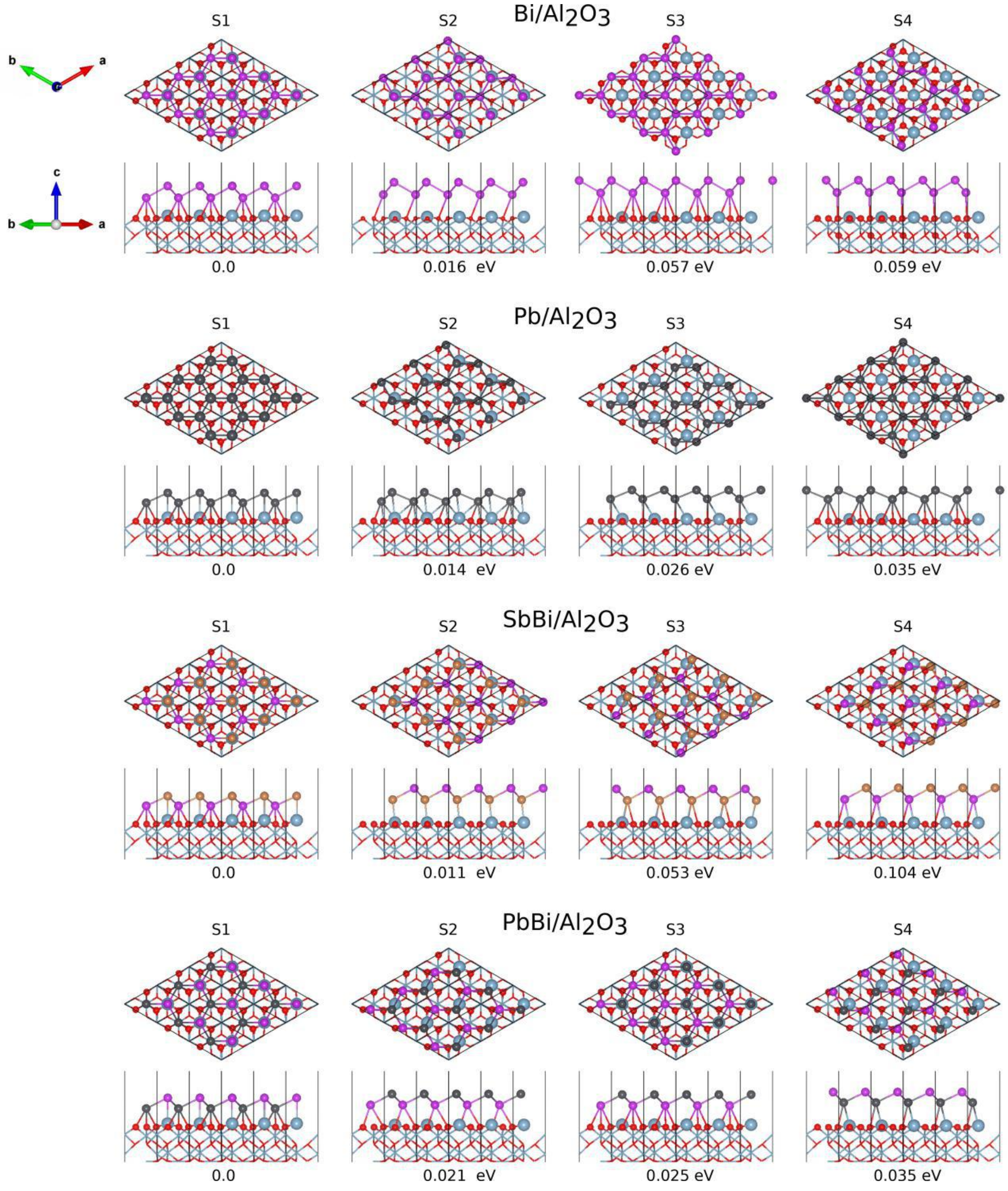

Figure S1. Top view and side view of the low-lying structures for the proposed systems. The energy differences are given relative to that for the lowest-energy structure S1.

2. Layer-dependence of the band structure for Pb/Al_2O_3(0001)

The band structure for Pb/Al_2O_3(0001) with an artificially increased layer-distance is plotted in Fig. S2.

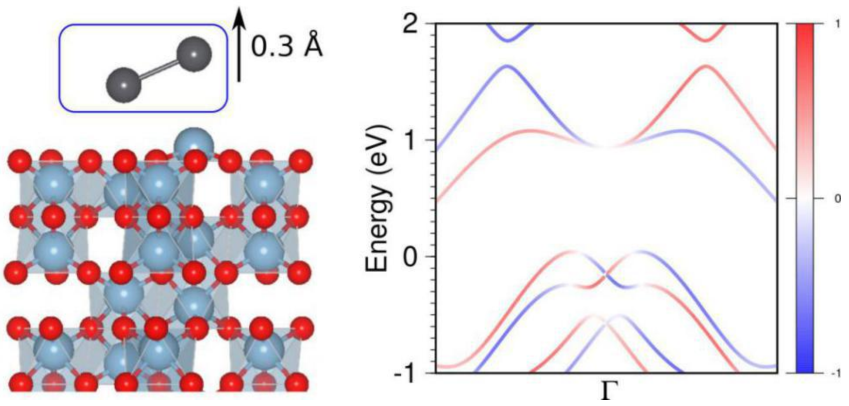

FIG. S2. Band structure for Pb/Al_2O_3(0001) with a layer-distance deviating from the equillibrium value. The layer distance between Pb the Al_2O_3(0001) is artistically increased by 0.3 Å.

3. Orbital-projected band structures for Bi/Al_2O_3(0001) and Pb/Al_2O_3(0001)

The orbital-projected band structures for Bi/Al_2O_3(0001) and Pb/Al_2O_3(0001) are plotted in Fig. S3.

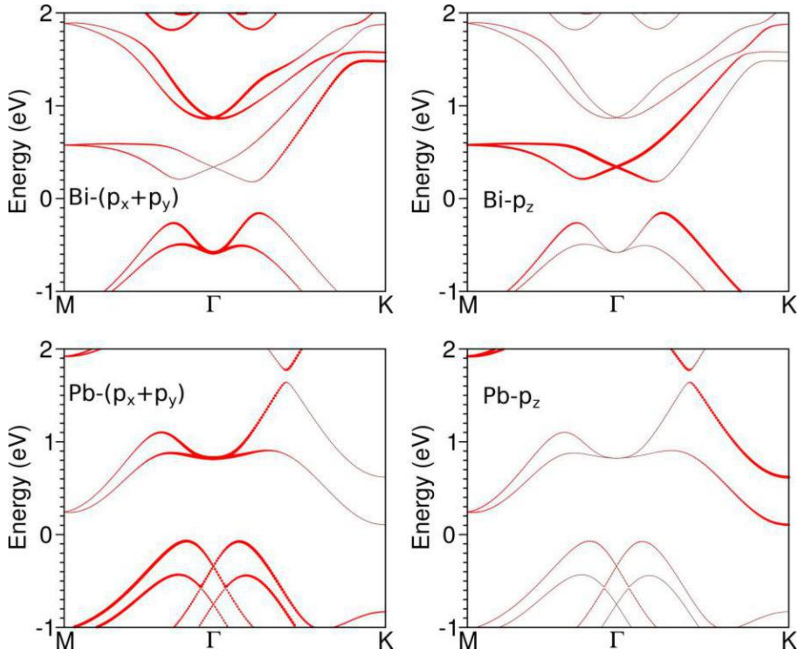

Fig. S3. Orbital-projected band structures for Bi/Al_2O_3(0001) and Pb/Al_2O_3(0001).

4. HSE06 calculation for SbBi/Al_2O_3(0001)

The HSE06 band structure of SbBi/Al_2O_3(0001) is shown in Fig. S4.

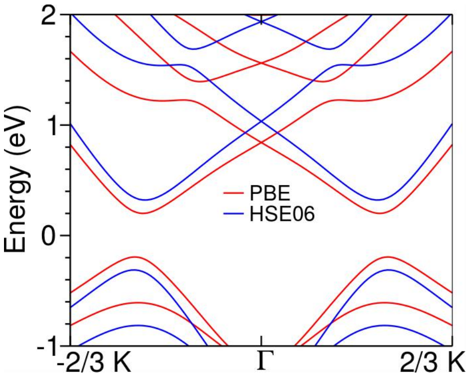

Fig. S4. HSE06 calculations for SbBi/Al_2O_3(0001). The PBE band structure is shown for comparison.

5. Band structures for $Sb_{1.1}Bi_{0.9}$/Al_2O_3(0001) and $Sb_{0.9}Bi_{1.1}$/Al_2O_3(0001)

The band structures of $Sb_{1.1}Bi_{0.9}$/Al_2O_3(0001) and $Sb_{0.9}Bi_{1.1}$/Al_2O_3(0001) are shown in Fig. S5.

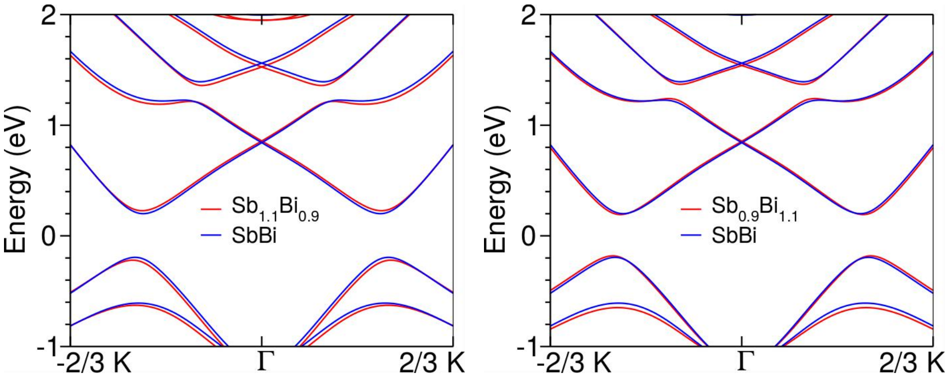

Fig. S5. The band structures of $Sb_{1.1}Bi_{0.9}$/Al_2O_3(0001) and $Sb_{0.9}Bi_{1.1}$/Al_2O_3(0001) using the virtual crystal approximation.

6. Effect of varying buckling height on the SO splitting in SbBi/Al_2O_3(0001)

The band structures of SbBi/Al_2O_3(0001) with the buckling ± 0.1 Å are shown in Fig. S6.

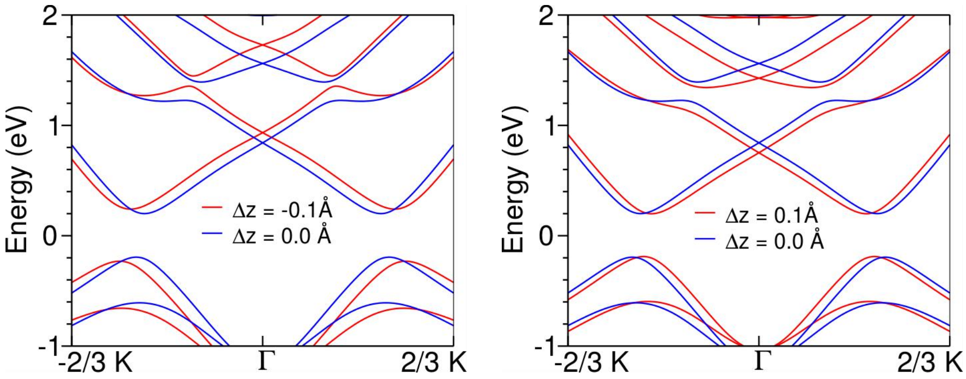

Fig. S6. The band structure of SbBi/Al_2O_3(0001) for the buckling height being reduced by 0.1 Å (left panel) and increased by 0.1 Å (right panel). The geometric structures corresponding

to the two bucklings are obtained by artificially moving the Sb closer to or further away from the substrate.

7. LCAO band structures for monolayers on $Al_2O_3(0001)$

The band structures for the proposed systems derived from LCAO calculations are plotted in Fig. S7.

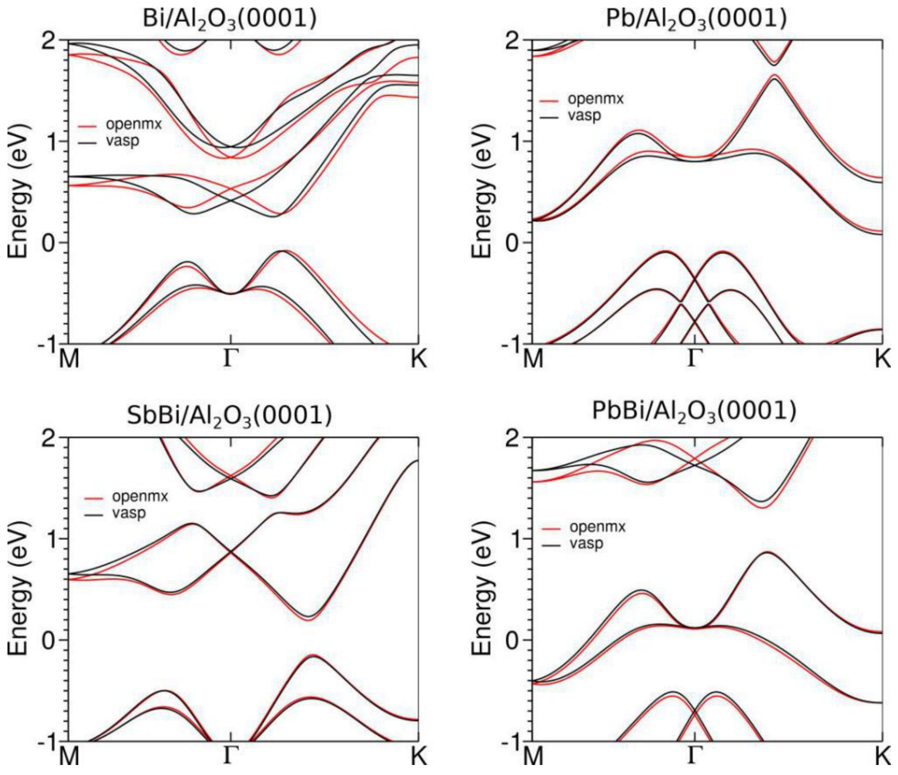

Fig. S7. LCAO band structures for monolayers on $Al_2O_3(0001)$.

8. Band structures for isolated SbBi and PbBi

The band structures for isolated SbBi and PbBi monolayers by removing the $Al_2O_3(0001)$ substrate are plotted in Fig. S8.

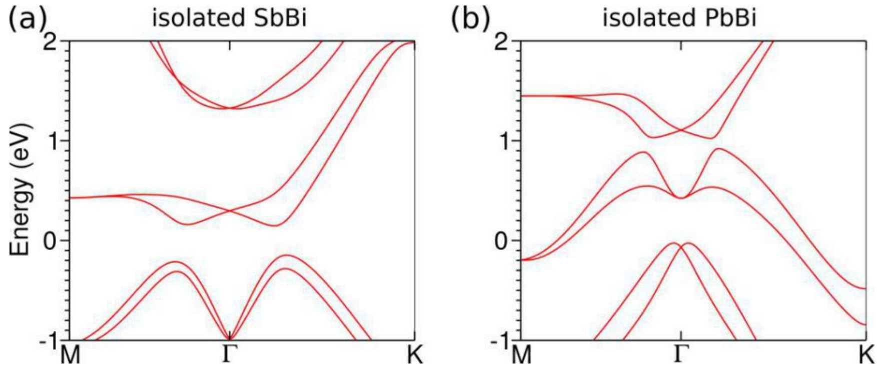

Fig. S8. Band structures for isolated SbBi and PbBi.

9. **Topological properties of monolayers on $Al_2O_3(0001)$**

Evolution of the Wannier function center and edge states for $Pb/Al_2O_3(0001)$ and $Bi/Al_2O_3(0001)$ are plotted in Fig. S9.

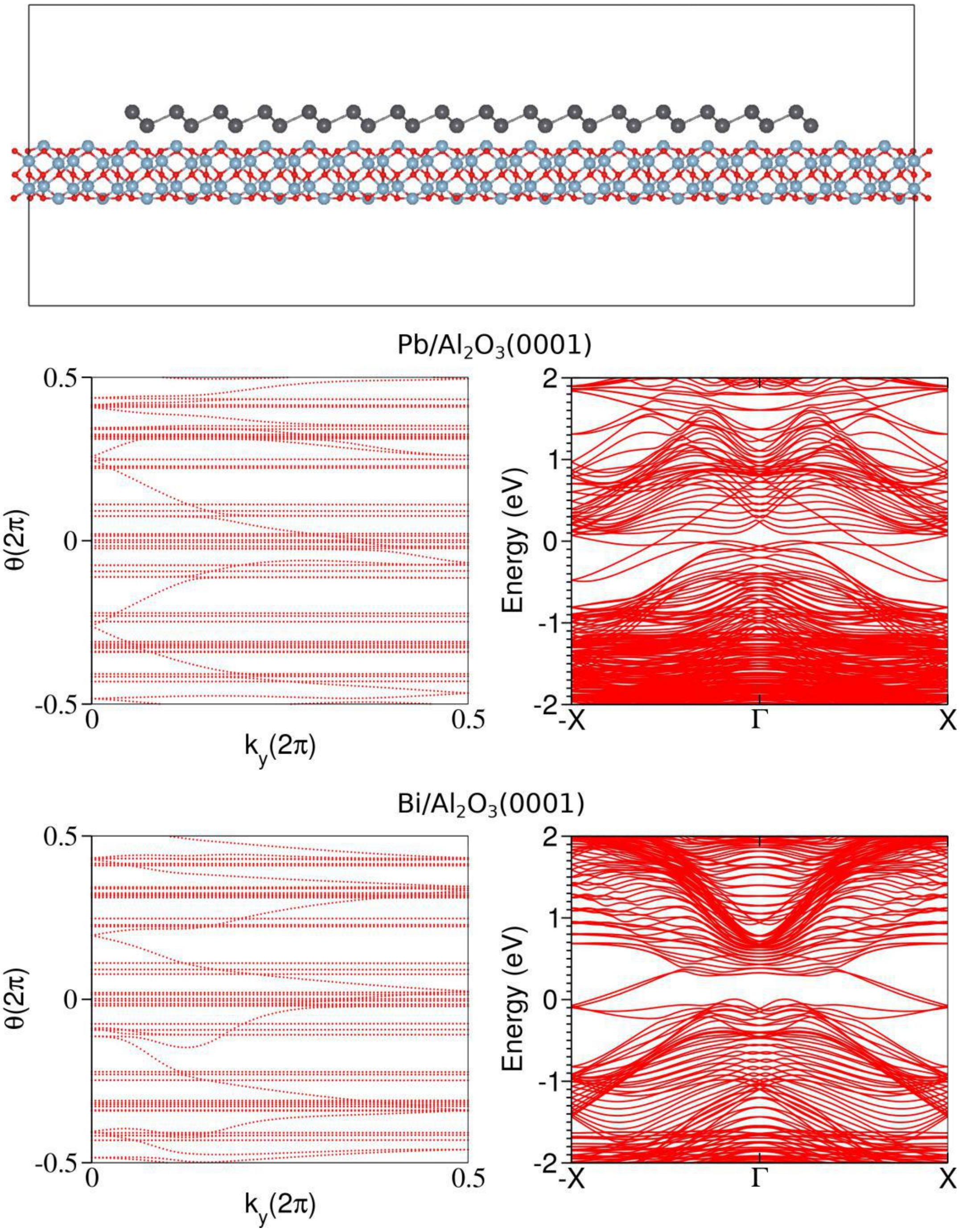

Fig. S9. Evolution of the Wannier function center and edge states for $Pb/Al_2O_3(0001)$, and $Bi/Al_2O_3(0001)$. The ribbon size for the calculation of edge states is about 6.4 nm.

10. *Ab initio* molecular dynamics simulation for SbBi/Al_2O_3(0001)

Results of *Ab initio* molecular dynamics simulation for SbBi/Al_2O_3(0001) are plotted in Fig. S10.

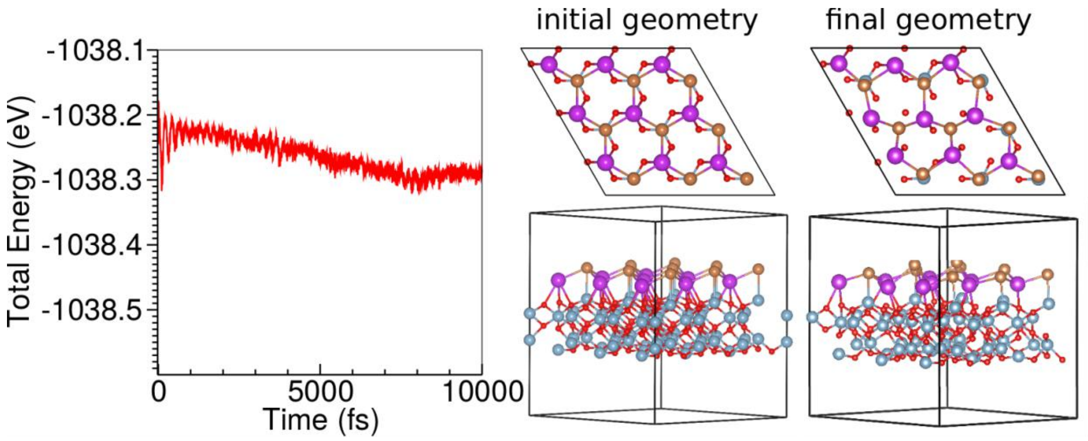

Fig. S10. *Ab initio* molecular dynamics simulation (T = 500K) for SbBi/Al_2O_3(0001).
